# Oral Cornstarch and Glycyrrhizin Improve Severe Liver Injury Caused by Glycogen Storage Disease Type IXa

**DOI:** 10.7759/cureus.78396

**Published:** 2025-02-02

**Authors:** Masanori Miyaishi, Kenji Fukushima, Naomi Kuranobu, Jun Murakami, Noriyuki Namba

**Affiliations:** 1 Division of Pediatrics and Perinatology, Faculty of Medicine, Tottori University, Tottori, JPN

**Keywords:** cornstarch, glycogen storage disease, glycyrrhizin, hepatomegaly, liver injury

## Abstract

A previously healthy 18-month-old boy presented with hepatomegaly, accompanied by liver injury. Imaging and liver biopsy findings suggested a hepatic glycogen storage disease (GSD) but not GSD type I. Genetic testing revealed a partial deletion of the PHKA2 gene, confirming the diagnosis of GSD type IXa. Initial treatment included ursodeoxycholic acid and portioned meals. However, the boy’s liver injury continued to worsen. Subsequently, nightly oral cornstarch after meals and oral glycyrrhizin were introduced. Following the addition of cornstarch and glycyrrhizin, the patient’s liver injury significantly improved. Liver injury caused by GSD is likely due to excessive glycogen accumulation in the hepatocytes. However, the detailed mechanism is unclear, particularly the minimal inflammatory cell infiltration observed in this case. While GSD IX has a good prognosis and resolves spontaneously, some patients develop hepatic fibrosis and adenomas. Cornstarch supplementation is a mainstay treatment for GSD to prevent hypoglycemia. It may also contribute to improved liver function by moderating glycogen buildup. The glycyrrhizin has also shown potential in reducing mitochondrial damage via another mechanism in this case, but additional research is warranted.

## Introduction

Hepatic glycogen storage disease (GSD) is a disorder characterized by fasting hypoglycemia, hepatomegaly, abdominal distention, doll-like facial features, and growth retardation. GSD type IX (GSD-Ⅸ) is a glycogen accumulation disease caused by a phosphorylase kinase (PhK) enzyme deficiency. PhK plays a crucial role in activating phosphorylase, an enzyme responsible for cleaving and releasing terminal glucose-1-phosphate units from the glycogen chain, thereby providing readily available glucose for cellular energy [1].

GSD-IX is divided into three clinical subtypes based on the specific defective enzyme, each associated with a different abnormal subunit. The American Society of Genetics and Genomics reports that approximately 75% of GSD-IX cases result from X-linked PHKA2 mutations (type IXa) (1). The phenotypes of GSD-IX vary, generally presenting as a liver injury with hepatomegaly and growth retardation between one and two years of age. Notably, these symptoms often resolve spontaneously over time [2]. The current situation in Japan does not allow for easy measurement of enzyme activity in hepatic GSD, so genetic testing is often used for definitive diagnosis.

We describe a case of a patient with GSD-IXa who presented with significant hypertransaminasemia that was successfully managed through dietary modifications and glycyrrhizin treatment.

## Case presentation

An 18-month-old boy was referred to our hospital with abdominal distention, progressive liver injury, and hepatomegaly detected on a computed tomography (CT) scan. His height was 74.3 cm (-3.3 standard deviation (SD)), and his weight was 10.8 kg (-0.5 SD). He displayed doll-like facial features. Abdominal examination revealed a 6 cm hepatomegaly with a smooth, elastic surface above the midclavicular line, with no palpable splenomegaly. Motor development was age-appropriate, and he had no history of seizures or altered consciousness. There was no family history of liver disease. 　

Laboratory investigations revealed hypochromic microcytic anemia, elevated liver enzymes (aspartate aminotransferase (AST), 630 IU/L; alanine aminotransferase (ALT), 432 IU/L; and hyperlactatemia, 53.2 mg/dL). He did not exhibit hypoglycemia, hypercholesterolemia, or elevated creatinine kinase levels (Table 1).

**Table 1:** Laboratory data on admission Alb, albumin; ALP, alkaline phosphatase; ALT, alanine transaminase; Apo AI, Apolipoprotein A-I; Apo B, Apolipoprotein B; AST, aspartate aminotransferase; BE, base excess; BS, blood sugar; BUN, blood urea nitrogen; ChE, cholinesterase; Cl, chloride; CPK, creatinine kinase; Cr, creatinine; CRP, C-reactive protein; D-BIL, direct bilirubin; Hb, hemoglobin; HCO3-, bicarbonate; Hct, hematocrit; K, potassium; LDH, lactate dehydrogenase; Na, sodium; NH3, ammonia; PAA analyses, plasma amino acid analyses; pCO2, partial pressure of carbon dioxide; PLT, platelet count; PT-INR, prothrombin time-international normalized ratio; RBC, red blood cell; T-BIL, total bilirubin; TC, total cholesterol; TG, triglyceride; TP, total protein; Urine Cu, urine cupper; WBC, white blood cell; γ-GTP, γ-glutamyl transferase.

Item	Result	Reference value (unit)
WBC	14,900	6.0-17.0 (/μL)
Hb	10.4	12.1-13.1 (g/dL)
Hct	33.2	36.5-39.5 (%)
PLT	58.3	15.8-34.8 (×10^4^/μL)
TP	6.1	6.6-8.1 (g/dL)
Alb	3.9	4.1-5.1 (g/dL)
T-Bil	0.5	0.4-1.5 (mg/dL)
D-Bil	0.1	< 0.4 (mg/dL)
AST	630	23-51 (U/L)
ALT	432	5-25 (U/L)
LDH	591	397-734 (U/L)
ALP	237	38-113 (U/L)
γ-GTP	144	13-64 (U/L)
ChE	193	240-486 (U/L)
CPK	62	59-248 (U/L)
TC	122	142-220 (mg/dL)
TG	266	40-149 (mg/dL)
NH_3_	49	12-66 (mg/dL)
BUN	16.3	6-20 (mg/dL)
Cr	0.36	0.3-0.6 (mg/dL)
CRP	0.05	< 0.15 (mg/dL)
BS	77	73-100 (mg/dL)
PT-INR	1.13	0.80-1.27
pH	7.396	7.35-7.45
pCO_2_	28.9	35-45 (mmHg)
HCO_3_^-^	17.3	22-26 (mmol/L)
BE	-6.6	-2 - +2 (mmol/L)
Lactate	44.9	3.0-17.0 (mg/dL)
Pyruvate	1.76	0.30-0.94 (mg/dL)
ceruloplasmin	23	21-37 (mg/dL)
Urine Cu	33	< 36 (µg/L)
Apo AI	129	119-155 (mg/dL)
Apo B	76	73-109 (mg/dL)
PAA analyses	No significant findings	

**Figure 1:**
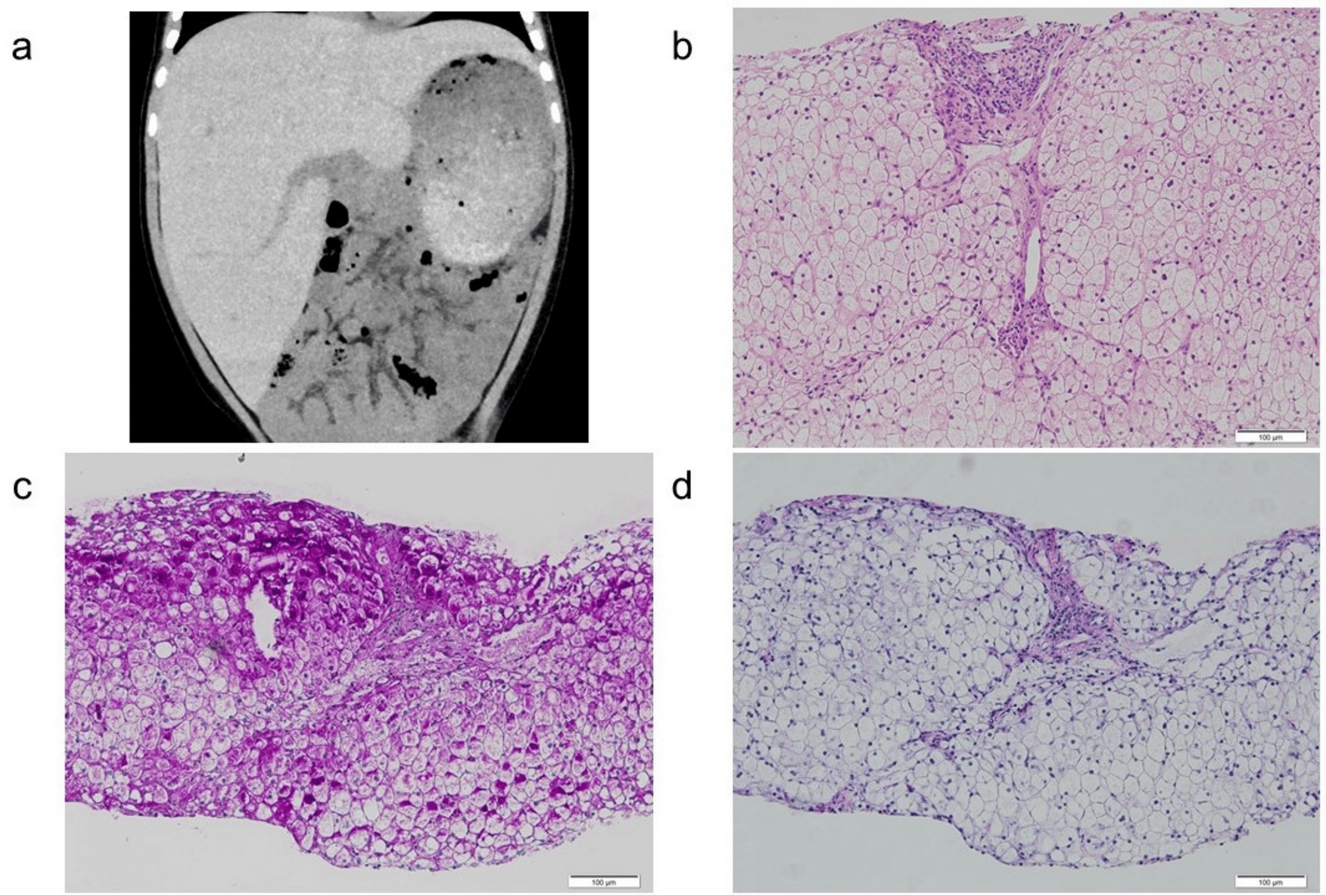
Abdominal imaging and liver histopathology (a) Abdominal plain computed tomography (CT) scan shows marked hepatomegaly with diffusely high density. (b) Hematoxylin and eosin (H&E) staining of liver biopsies reveals vacuolar degeneration within hepatocytes (original magnification: ×100). (c) Periodic acid-Schiff (PAS) staining highlights intracellular glycogen accumulation within hepatocytes (original magnification: ×100). (d) Periodic acid-Schiff with diastase (PAS-D) staining confirms the presence of glycogen by showing a reduction in positive staining compared with a panel.

**Figure 2:**
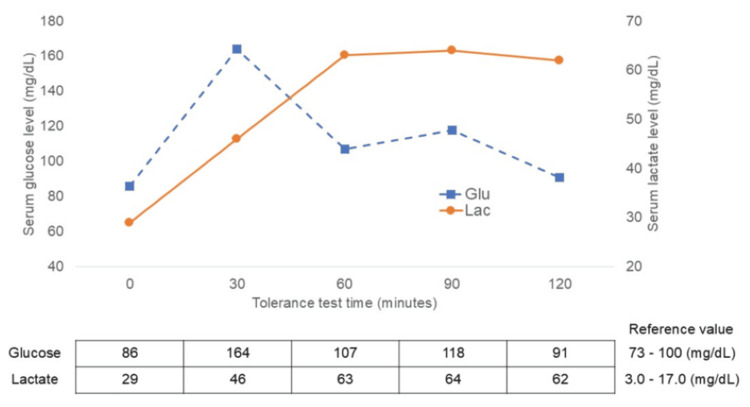
Oral glucose tolerance test The oral glucose tolerance test shows that our patient had increased serum lactate level after glucose intake.

Abdominal ultrasonography showed marked hepatomegaly with diffuse high echogenicity and CT scan showed high density. The patient showed no signs of splenomegaly (Figure 1a). Echocardiography revealed no myocardial hypertrophy or dysfunction. Further, a percutaneous liver biopsy revealed vacuolar degeneration of hepatocytes, hepatocellular glycogen accumulation, and microvesicular steatosis (Figures 1b, 1c, 1d). A glucose tolerance test revealed a transient increase in lactate levels without postprandial hypoglycemia (Figure 2). Subsequently, next-generation sequencing analysis identified a deletion encompassing exons 3-4 of PHKA2, confirming the diagnosis of GSD IXa.

**Figure 3:**
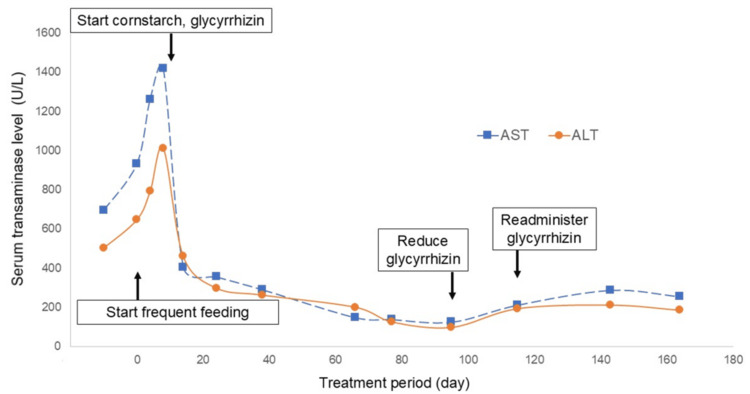
Clinical course of liver injury This illustrates the changes in liver function tests over time. A significant decrease in transaminase levels (AST and ALT) is observed after initiating oral cornstarch and glycyrrhizin therapy. Following discontinuation of glycyrrhizin, transaminase levels worsen, only to improve again upon reintroduction of glycyrrhizin.

Imaging and histological examinations strongly suggested that he had hepatic GSD. The glucose tolerance test results excluded GSD type I but did not definitively rule out GSD types III, VI, and IX. Following the diagnostic criteria of the Japanese Society for Inherited Metabolic Diseases, he was diagnosed with a suspected case of hepatic-type GSD other than GSD-Ⅰ prior to genetic screening. After obtaining informed consent for GSD, treatment for liver injury was initiated with ursodeoxycholic acid, a hepatoprotective agent. However, it resulted in worsening of liver function. The patient was placed on a frequent feeding regimen (six small meals per day, 45 g rice per meal) to prevent glycogen accumulation and received oral bicarbonate to address metabolic acidosis. Despite these treatments, his liver injury was further aggravated, with AST and ALT levels reaching 1,420 IU/L and 1,011 IU/L, respectively. Additionally, low hemoglobin A1c (HbA1c, 4.7 %) suggested potential subclinical hypoglycemia, prompting initiation of nightly cornstarch supplementation after meals to sustain blood sugar levels. Glycyrrhizin, a medication with reported hepatoprotective properties, was also administered orally (12.5 mg per dose, three times a day). Within a few days, liver function tests and hemoglobin A1c started improving and the patient was discharged with AST and ALT levels of 405 IU/L and 459 IU/L, respectively. Following discharge, discontinuation of glycyrrhizin resulted in a resurgence of liver injury, necessitating its readministration. The patient’s clinical course is depicted in Figure 3.

## Discussion

GSD-IX typically shows a favorable prognosis. However, our case presented with severe liver injury, highlighting the variable clinical course of this disease. Existing literature reports cases of GSD-IX with substantial liver injury [3,4]. Liver injury caused by GSDs is thought to be due to excessive glycogen accumulation in hepatocytes. However, the detailed mechanism remains unclear. Mitochondrial dysfunction and apoptotic pathway activation have been observed in both in vitro and in vivo models of GSD [5]. This mitochondrial dysfunction and apoptosis induction may exacerbate liver injury progression. Notably, liver histopathology of glycogenic types VI and IX, including our patient, demonstrates minimal inflammatory cell infiltration [6]. Furthermore, some patients with GSD-IX develop hepatic fibrosis and/or hepatocellular adenomas. Since apoptosis induces fibrosis [7], early management of liver injury in GSD may be important for preventing future complications.

Appropriate nutritional therapy for patients with GSD is critical to improving their condition [8]. On the other hand, the current literature on GSD treatment primarily focuses on preventing hypoglycemia, with limited information on managing liver injury. Dietary therapies, such as restriction of sucrose, fructose, and lactose, along with scheduled uncooked cornstarch dosing, are recommended to prevent hypoglycemia in GSDs [9]. In our case, the patient’s low HbA1c level suggested subclinical hypoglycemia, prompting the initiation of cornstarch to prevent hypoglycemia. Following oral cornstarch administration, the patient’s HbA1c gradually improved. Cornstarch is gradually broken down into glucose by α-amylase, thus suppressing the temporary excessive rise in blood glucose and the rapid accumulation of glycogen [10]. This mechanism indicates that cornstarch prevents hypoglycemia and potentially mitigates liver injury by moderating glycogen accumulation.

Furthermore, oral glycyrrhizin administration demonstrated efficacy in improving liver function in our patient. Glycyrrhizin, an active ingredient extracted from licorice root, is commonly used to reduce liver inflammation. In Japan, the dosage of oral glycyrrhizin is stated in the package insert as 25 mg per dose, three times a day in pediatric patients with chronic liver disease. Adverse effects of this drug include pseudohypoaldosteronism and rhabdomyolysis. Studies have shown that glycyrrhizin increases interleukin (IL)-25 levels, a potential mechanism for its ability to ameliorate liver injury [11]. IL-25 has been reported to have anti-inflammatory effects by inhibiting T-helper (Th)17 cell differentiation through the PI3K/Akt pathway, reducing IL-17 production and inducing a regulatory T-cell phenotype [12,13]. Moreover, glycyrrhizin has been shown to reduce mitochondrial damage by inhibiting neuronal nitric oxide synthase upregulation in acetaminophen-induced liver injury [14] and to reduce liver injury by inhibiting tissue necrosis factor (TNF)α-dependent apoptosis [15]. In our case, the potential benefits of glycyrrhizin may lie in its ability to mitigate mitochondrial dysfunction and apoptosis rather than solely through its anti-inflammatory effects. It is possible that cornstarch, started at the same time as glycyrrhizin, may have influenced the outcome. However, in this case, liver damage worsened after discontinuing glycyrrhizin and improved after resuming the drug. This suggests that glycyrrhizin was effective against liver damage.

## Conclusions

Our case in GSD-IXa had severe hepatic injury. This hepatic injury is thought to be caused by the rapid accumulation of glycogen in hepatocytes, and it is important to control the diurnal variation of blood glucose levels to prevent this hepatic injury. Cornstarch can be used for this purpose. In addition, orally administered glycyrrhizin may reduce liver damage by inhibiting mitochondrial dysfunction and apoptosis. Reducing liver damage with these treatments may also reduce the risk of future liver fibrosis. Further case accumulation and patient follow-up are needed to confirm the efficacy of these treatments.
